# Catheter-Based Radiofrequency Renal Sympathetic Denervation Decreases Left Ventricular Hypertrophy in Hypertensive Dogs

**DOI:** 10.1155/2021/9938486

**Published:** 2021-04-24

**Authors:** Shan Tu, Zhi-Jie Shen, Xiao-Yan Wang, Li-Xiong Zeng, Zhi-Hui Zhang

**Affiliations:** Department of Cardiology, The Third Xiangya Hospital of Central South University, Changsha, 410006 Hunan Province, China

## Abstract

This study explored the effects of renal sympathetic denervation (RDN) on hyperlipidity-induced cardiac hypertrophy in beagle dogs. Sixty beagles were randomly assigned to the control group, RDN group, or sham-operated group. The control group was fed with a basal diet, while the other two groups were given a high-fat diet to induce model hypertension. The RDN group underwent an RDN procedure, and the sham-operated group underwent only renal arteriography. At 1, 3, and 6 months after the RDN procedure, the diastolic blood pressure (DBP) and systolic blood pressure (SBP) levels were markedly decreased in the RDN group relative to the sham group (*P* < 0.05). After 6 months, serum norepinephrine (NE) and angiotensin II (AngII), as well as left ventricular levels, in the RDN group were statistically lower than those in the sham group (*P* < 0.05). Also, the left ventricular mass (LVM) and left ventricular mass index (LVMI) were significantly decreased, while the E/A peak ratio was drastically elevated (*P* < 0.05). Pathological examination showed that the degree of left ventricular hypertrophy and fibrosis in the RDN group was statistically decreased relative to those of the sham group and that the collagen volume fraction (CVF) and perivascular circumferential collagen area (PVCA) were also significantly reduced (*P* < 0.05). Renal sympathetic denervation not only effectively reduced blood pressure levels in hypertensive dogs but also reduced left ventricular hypertrophy and myocardial fibrosis and improved left ventricular diastolic function. The underlying mechanisms may involve a reduction of NE and AngII levels in the circulation and myocardial tissues, which would lead to the delayed occurrence of left ventricular remodeling.

## 1. Introduction

The rapid emergence and serious consequence of hypertension are now considered one of the major threats to the quality of human life. Hypertension is not only a risk factor for myocardial infarction, coronary artery disease, and stroke but also the most common cause of death [1, 2]. Refractory hypertension is a vital risk factor for the occurrence of cardiovascular events. Patients with hypertension experience changes in left ventricular hypertrophy (LVH) and myocardial fibrosis, even before the incidence of clinical events [3].

Structural cardiac changes are often accompanied by impaired left ventricular function, such as abnormal left ventricular relaxation and increased left ventricular diastolic filling pressure. Studies have shown that diastolic dysfunction may occur in hypertensive subjects with a normal left ventricular mass (LVM) [4, 5]. Left ventricular hypertrophy and diastolic dysfunction are closely related to death and disability rates for cardiovascular diseases [6, 7]. Studies have found that the reversal of LVH improves the prognosis of cardiovascular diseases and that LVH is independent of other risk factors. Therefore, LVH is recommended as an intermediate treatment endpoint [8].

As a novel technology for treating refractory hypertension, catheter-based radiofrequency renal sympathetic denervation (RDN) decreases the activity of the afferent and efferent renal sympathetic nerve fibers through catheter ablation at the vascular intima, thereby effectively lowering diastolic blood pressure (DBP) and systolic blood pressure (SBP) in cases of refractory hypertension and reducing the heart rate of patients with refractory hypertension [9–11]. In addition, Mahfoud et al. demonstrated that catheter-based RDN significantly decreased the level of BP and left ventricular mass index (LVMI), resulting in improved ejection fraction (EF) and circumferential strain in subjects with resistant hypertension [12]. In subjects for whom the office-based blood pressure exhibits a slight decrease of less than 10 mmHg at 6 months after the RDN procedure, RDN reduces LVM. The above effects of RDN may be independent of effects such as decreased blood pressure and the lowering of the heart rate [13, 14].

Due to the limitations of RDN, such as unclear mechanisms of action, imperfect surgical methodology, and nonideal surgical instruments, there is a great deal of scientific controversy regarding RDN. The effects of RDN on the structure and function of the left ventricular and the vital factors associated with the long-term prognosis of patients with hypertension require further investigation. In this study, we explored the changes in blood pressure level, the severity of LVH, and the diastolic function of the left ventricular in hypertensive dogs treated with RDN.

## 2. Materials and Methods

### 2.1. Experimental Animals

Sixty beagles, half of which were males and half of which were females, were bought from the Shanghai Laboratory Animal Center. All the dogs were 10–12 months of age and belonged to the same strain. Dogs were housed individually in separate cages at the Xiangya Animal Facility of Central South University. Then, after adaptive feeding for 7 days, the dogs underwent bilateral renal arteriography to determine their renal arterial anatomy. All experimental protocols were approved by the Animal Care and Use Committee of the Third Xiangya Hospital of Central South University and performed in accordance to the guidelines of Animal Care and Use Committee of the Third Xiangya Hospital of Central South University.

### 2.2. Establishment of the Hypertension Model

The sixty beagles were randomly assigned to the control group, the RDN group, or the sham-operated group (*n* = 20, individually). In the control group, the animals were given a diet formulated for adult dogs (250 g of food per dog per day). The RDN group and the sham-operated group were fed with a high-fat diet for 90 days (adult dog food [150 g/day] + lard [0.4 kg/day]). The edible lard was bought from the market (Green Island [Sichuan] Food Co., Ltd.). To stimulate the dogs' appetites, an appropriate amount of salt and canned dog food was added to the diets of both groups. The dogs were fed regularly once per day between 9:00 and 10:00 a.m. and were given a dog calcium supplement (1 tablet) every other day. After 3 months of high-fat feeding, one beagle in the RDN group and one in the sham-operated group had blood pressure levels that did not meet the criteria for hypertension, indicating a failure to establish the hypertension model. Therefore, the corresponding data were excluded. After the RDN procedure, the dogs in the RDN group and the sham-operated group were given a high-fat diet to maintain a constant body mass.

### 2.3. Monitoring Body Weight, Blood Pressure Level, and Heart Rate

All dogs were weighed regularly at 9:00 a.m. under fasting conditions using electronic scales. Using a noninvasive animal blood pressure meter (Beijing Ruanlong Biotechnology Co., Ltd.), the blood pressure level in the tail arterial of the beagles was measured while the dogs were fully awake. Beagles were restrained in animal holders. Once a beagle became completely calm, cuffs were placed on the roots of the dogs' tails. After setting up the correct parameters, the blood pressure monitors automatically read the SBP level, DBP level, and heart rate value. Each dog was measured six times. Data that deviated from the expected standard error were excluded, and the means of the remaining data were calculated. Before the experiment, blood pressure levels and heart rates of the beagles were recorded twice per week. The preparation of the hypertension model began once the blood pressure and heart rate data became stable, which were used as the baselines. The blood pressure level and heart rate were detected at the following time points: before the protocol of the hypertension model and then at 2 weeks, 4 weeks, 6 weeks, and 12 weeks. The RDN group was subjected to the RDN procedure. The blood pressures and heart rates of all dogs were measured at 1, 3, and 6 months after the RDN treatment.

### 2.4. Echocardiography

Color Doppler echocardiography was conducted on the anesthetized experimental dogs prior to renal arteriography using the Toshiba 880 cardiac ultrasound machine. Echocardiography was performed at baseline, before the RDN procedure, and after the RDN procedure by the same cardiac sonographer. The end-diastolic diameter of the interventricular septum (IVSd), left ventricular end-diastolic diameter (LVEDD), and left ventricular posterior wall dimension (LVPWd) were detected using M-type ultrasound. The LVM was calculated using the Devereux equation. In addition, the left atrial diameter, ejection fraction (EF) value, and peak E/A ratio were determined.

### 2.5. Examination of the Biochemical Indices

The experimental dogs were restrained in holders. Blood samples were collected from the small saphenous vein located on the lateral side of the hind limbs of the dogs in the morning under fasting conditions. Samples were processed and tested in accordance with the corresponding requirements. The kidney tissues were collected at 6 months after the RDN procedure. The following biochemical indices were examined: serum creatinine (measured using the chemiluminescence method), cystatin C (using the immune transmission turbidity method), kidney norepinephrine (NE, using high-performance liquid chromatography [HPLC]), and angiotensin II (AngII) (using enzyme-linked immunosorbent assay [ELISA] and radioimmunoassay).

### 2.6. RDN Procedure

The RDN group underwent the RDN procedure, and the sham-operated group received only renal arteriography. Beagles were fasted before the procedure. The skin of the back and the bilateral femoral artery area was prepared. Each beagle was anesthetized via an intramuscular injection of 15 mg/kg ketamine, followed by the inhalation of 5% isoflurane for 3 min. After oral tracheal intubation, the inhalation isoflurane concentration was changed to 2% until 10 min before surgery. After anesthesia, the animals were immobilized in the supine position. Ablation electrodes were placed on the back of the dogs and connected to a radiofrequency ablation device (IBI, St. Jude Medical, Inc., USA). The temperature was maintained at around 55°C, and the ablation power was set at 10 W. The area of the right femoral artery was cleaned with conventional disinfection techniques. Then, the right femoral artery was punctured, and an 8F arterial sheath was inserted into the femoral artery. One hundred U/kg heparin was injected along the sheath. Subsequently, a guiding catheter was inserted into the arterial sheath while the blood pressure level was monitored. Afterward, renal arteriography was conducted, and the renal artery was located. A standard 5F radiofrequency ablation catheter was then inserted in the arterial sheath (IBI radiofrequency ablation catheter, St. Jude Medical, Inc., USA). Four spots on each side of the inner wall of the renal artery were selected as the ablation sites, and sequential point-by-point ablation was performed in the order of lower wall, anterior wall, posterior wall, and upper wall [15]. Each site was ablated continuously for 120 s. After successful bilateral renal artery ablation, renal arteriography was performed once again to determine whether renal artery dissection or stenosis had occurred. Finally, the sheath was removed. Sterile gauze was pressed against the puncture site for 45 min to stop the bleeding. The right hind limb was then bandaged. Penicillin was administered via intramuscular injection to prevent infection. At 1, 3, and 6 months after the RDN procedure, the dogs were reexamined and subjected to renal arteriography.

### 2.7. Euthanasia and Tissue Collection

Six months after the RDN procedure, the animals were sacrificed via an intravenous injection of 10 ml saturated KCl after being anesthetized by the inhalation of isoflurane (5%) for 10 min. The hearts were collected, and the NE and AngII content in the left ventricular was determined. In addition, the left ventricular tissues were fixed and used for hematoxylin and eosin (HE) staining and Masson's trichrome staining. Subsequently, the pathological structural changes in the left ventricular tissues were analyzed via microscopy and electron microscopy. The cardiac tissues stained with Masson's trichrome were further analyzed using a light microscopy and image analysis system to determine the myocardial collagen volume fraction (CVF) and myocardial perivascular circumferential collagen area (PVCA). The CVF was calculated with the following formula: CVF = collagen area/total area of each microscopic field of view. Five randomly selected fields of view were analyzed, and the cardiac CVF was expressed as the mean CVF of the five fields. The PVCA was calculated using the following formula: PVCA = area of collagen surrounding the arterioles in the ventricular wall/arterial lumen area. In each sample, four arterioles in the ventricular wall were selected, and the cross section of the arterioles was analyzed. The mean PVCA was considered the final cardiac PVCA.

### 2.8. Statistical Analysis

The data analysis was conducted using SPSS 19.0 software. Most data were shown as the means ± standard deviations (SD), whereas the count data were presented as the incidence rate or constitute ratio. All data were analyzed with a normality test and homogeneity-of-variance test. If the data were normally distributed and met the assumption of homogeneity of variances, the comparison of the means between the two groups was performed with Student's *t*-test, and the comparison between multiple groups was conducted using a one-way analysis of variance (ANOVA). If the data failed to show homogeneity of variance, the comparison between the two groups was analyzed with the Mann-Whitney test, whereas the comparison between multiple groups was analyzed with the Kruskal-Wallis test. The comparison of the heart rate was performed with Fisher's exact test. A *P* value of less than 0.05 was considered statistically significant.

## 3. Results

### 3.1. Safety of the RDN Procedure

No deaths occurred in the experiment; ten dogs had hematomas at the puncture sites. After reapplying pressure and bandages, the dogs suffered no sequelae. During the ablation procedure, five dogs experienced renal artery spasm. These dogs were given nitroglycerin injections. Approximately 10 min after the injection, renal artery spasm was alleviated. Renal angiography was conducted immediately after the RDN procedure. No renal artery dissection or thrombosis was detected. The dogs were reexamined at 1, 3, and 6 months after the RDN procedure, and renal arteriography showed no evidence of renal artery stenosis.

### 3.2. Changes in Body Weight, Heart Rate, Blood Lipid Level, Renal Function, and Blood Pressure in the Experimental Dogs (Table 1, Figure 1)

As shown in Table 1, no statistical differences were found in baseline body weight, heart rate, blood lipid level, creatinine level, cystatin C level, or blood pressure level between the three groups (*P* > 0.05). After the establishment of the hypertension model (before the RDN procedure), the body weight, low-density lipoprotein (LDL) level, total cholesterol (TC) level, triglyceride (TG) level, SBP level, and DBP level were significantly increased in the animals of the RDN group and the sham-operated group relative to the baselines and those of the control group (*P* < 0.05). Meanwhile, no statistical differences were detected in the above indicators between the RDN group and sham-operated group (*P* > 0.05). Heart rate was slightly increased in the RDN group and the sham-operated group after the establishment of the model relative to the baseline and the control group (*P* > 0.05). The body weights of animals in the RDN group and the sham-operated group also were not significantly increased after the RDN procedure relative before the RDN procedure (*P* > 0.05). Heart rate was slightly decreased in the dogs in the RDN group at 1, 3, and 6 months after the RDN procedure. However, no statistical differences were detected in heart rates before and after the RDN procedure (*P* > 0.05). At 1, 3, and 6 months after the RDN procedure, the heart rate was slightly raised in the RDN group and the sham-operated group relative to the control group (*P* > 0.05). The creatinine and cystatin C levels in the animals of the RDN group were slightly different from those before the RDN procedure at 1, 3, and 6 months after the RDN procedure. In addition, there were no significant differences in creatinine and cystatin C levels between the RDN group and sham-operated group or between the RDN group and the control group at the same time points after the RDN procedure (*P* > 0.05).

The SBP and DBP levels were markedly decreased in the RDN group at 1, 3, and 6 months after the RDN procedure. At each time point after the RDN procedure, the SBP and DBP levels were decreased in the RDN group relative to those of the sham-operated group (*P* < 0.05). After the RDN procedure, the RDN group had similar blood pressure as compared to that in the control group. The difference in blood pressure level was not significant (*P* > 0.05). No statistical changes were detected in the levels of SBP and DBP in the sham-operated group at various time points after the procedure (*P* > 0.05) (Table 1, Figure 2).

### 3.3. Changes in Kidney NE and AngII in the Experimental Dogs

**Figure 1:**
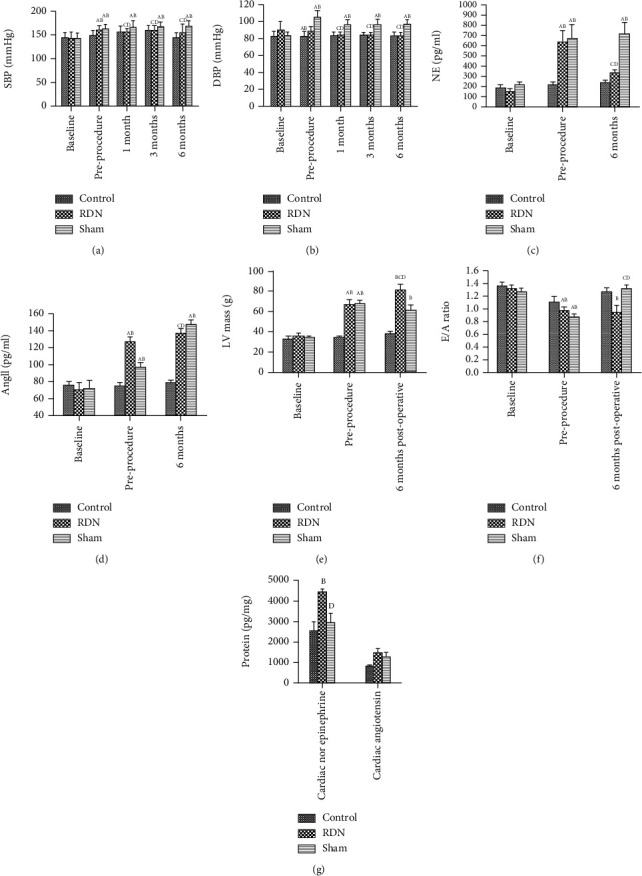
Changes in blood pressure, left ventricular mass (LVM), peak E/A ratio, norepinephrine (NE), and angiotensin II (AngII) in experimental dogs. ^A^Compared with baseline, *P* < 0.05. ^B^Compared with the control group, *P* < 0.05. ^C^Compared with the sham group, *P* < 0.05. ^D^Compared with preprocedure, *P* < 0.05.

**Figure 2:**
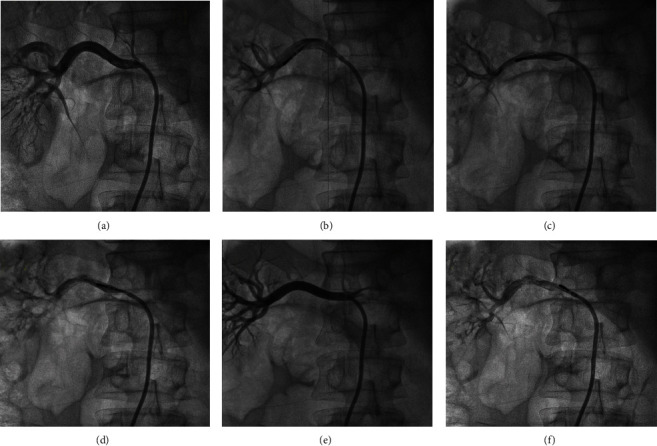
RDN procedure: (a) right renal arteriography before ablation; (b) ablation of lower wall of the right renal artery; (c) ablation of anterior wall of the right renal artery; (d) ablation of posterior wall of the right renal artery; (e) ablation of upper wall of the right renal artery; (f) right renal arteriography after ablation.

As shown in Table 2 and Figure 1, no statistical differences were found in the levels of kidney NE and AngII between the control group, RDN group, and sham-operated group (*P* > 0.05). After the establishment of the hypertension model, the levels of kidney NE and AngII were drastically increased in the RDN group and sham-operated group in comparison with the baselines and the control group (all *P* < 0.05). No statistical differences were found in the serum kidney NE and AngII levels between the RDN group and the sham-operated group (*P* > 0.05). Compared with baseline, the level of serum AngII was slightly elevated in the control group after the establishment of the model (*P* > 0.05). After 6 months of the RDN procedure, the serum levels of NE and AngII were markedly decreased in the RDN group relative to the NE and AngII levels before the procedure and in the sham-operated group after the procedure (*P* < 0.05). After 6 months of the RDN procedure, the levels of NE and AngII in the left ventricular myocardial tissues were drastically decreased in the RDN group relative to the sham-operated group, whereas the AngII level was higher in the RDN group relative to the control group (all *P* < 0.05). In addition, the sham-operated group had significantly higher levels of NE and AngII in the left ventricular myocardial tissues relative to the control group (*P* < 0.05).

### 3.4. Echocardiographic Evaluation of Structural Cardiac Changes in the Experimental Dogs

As shown in Table 3, no statistical differences were detected in baseline LVM, LVMI, and peak E/A ratio (*P* > 0.05) between the three groups. After the establishment of the model, the LVM and LVMI were drastically increased, while the peak E/A ratio was statistically reduced, in the RDN group and the sham-operated group relative to the baselines and the control group (*P* < 0.05). At 6 months after the RDN procedure, LVM and LVMI were statistically lower in the RDN group relative to the sham-operated group (*P* < 0.05), whereas the peak E/A ratio was drastically increased in the RDN group relative to the sham-operated group (*P* < 0.05).

### 3.5. Pathological Changes in the Left Ventricular Myocardium of the Experimental Dogs

**Figure 3:**
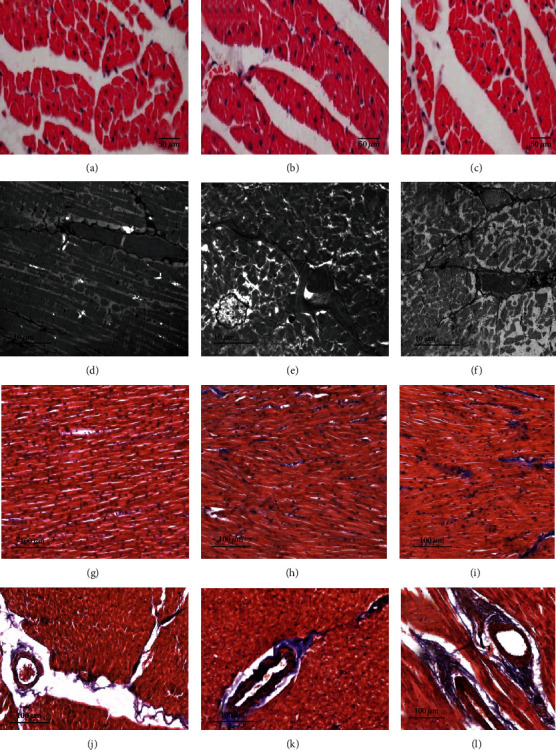
Pathological changes in the left ventricular myocardium of experimental dogs. (a–c) HE staining of myocardial tissue (light microscopy, 400x): (a) normal myocardial tissue in the control group, (b) the RDN group, and (c) the sham-operated group. The size and cross-sectional area of cardiomyocytes were statistically smaller in the RDN group than in the sham-operated group. Additionally, cardiomyocytes in the RDN group were arranged in a more orderly manner, similar to the control group. (d–f) Electron microscopy images of myocardial tissue (12000x): (d) the control group, (e) the RDN group: a portion of cardiomyocyte myofilaments was dissolved and ruptured, and a fraction of the mitochondria became swollen, and (f) the sham-operated group: multifocal dissolution and fragmentation of the myofilaments were apparent, mitochondrial and cellular swelling was evident, and the numbers of the mitochondria and cells were markedly reduced. (g–i) Masson's trichrome staining of myocardial tissue (light microscopy, 400x): (g) the control group, (h) the RDN group: a small number of collagen fibers were deposited in the myocardial stroma, and (i) the sham-operated group: large numbers of collagen fibers were produced in the myocardial stroma (red indicates cardiomyocytes, blue indicates collagen fibers, and red arrow points to collagen fiber). (j–l) Masson's trichrome staining of myocardial tissue (light microscopy, 400x): (j) the control group, (k) the RDN group: a small number of collagen fibers were deposited in the perivascular area, and (l) the sham-operated group: large numbers of collagen fibers were produced in the perivascular area (red indicates cardiomyocytes, blue indicates collagen fibers, and red arrow points to collagen fiber).

The left ventricular myocardium was subjected to H&E staining, and the results showed that the size and the cross-sectional area of the cardiomyocytes were statistically smaller in the RDN group relative to the sham-operated group (Figure 3). In addition, the cardiomyocytes in the RDN group were arranged in a more orderly manner. Electron microscopy revealed that the myocardial myofilaments were intact in the RDN group. Only a portion of the myofilaments in the cardiomyocytes were dissolved and ruptured. A fraction of the mitochondria and cells became swollen. By contrast, multifocal dissolution and fragmentation of the myofilaments were found in the sham-operated group. In the sham-operated group, mitochondrial and cellular swelling was evident, and the numbers of mitochondria and cells were statistically reduced. Masson's trichrome staining revealed that a small number of collagen fibers were deposited in the myocardial stroma and perivascular area in the RDN group, whereas a large number of collagen fibers were produced in the sham-operated group. As shown in Table 4, the Masson staining of the myocardium demonstrated that CVF was statistically increased in the sham-operated group and the RDN group relative to the control group (*P* < 0.05). In contrast, the CVF was statistically decreased in the RDN group relative to the sham-operated group (4.32 ± 1.33% vs. 12.65 ± 1.67%, *P* < 0.05). The PVCA was statistically increased in the sham-operated group relative to the control group (*P* < 0.05). No statistical differences in PVCA were detected between the RDN group and the control group (*P* > 0.05). In addition, PVCA was markedly decreased in the RDN group relative to the sham-operated group (0.85 ± 0.16 vs. 1.11 ± 0.19, *P* < 0.05).

## 4. Discussion

In the present study, we found that an RDN procedure not only effectively decreased the blood pressure levels in hypertensive dogs induced via a high-fat diet but also reduced left ventricular hypertrophy and myocardial fibrosis and improved the left ventricular diastolic function. The functional improvement (diastolic function) of RDN has potential implications for heart failure, which is another area of clinical interest as a potential application for RDN [16].

A high-fat diet is now widely used to establish models of hypertension [17]. Specifically, neurohormonal and hemodynamic changes in the high-fat diet-induced hypertension model are virtually the same as the pathophysiological changes in human hypertension. Moreover, the high-fat diet-induced model is more likely to display LVH in the early stages of model development. According to previous studies, the construction of the hypertension model is considered successful if blood pressure levels in the dogs are measured at 17 ± 2 mmHg over the baseline after high-fat feeding [18]. In our research, after 3 months of high-fat feeding, the dogs exhibited statistically increased body weight and TC and LDL levels relative to the control group. The SBP level increased by more than 20 mmHg in 90.9% (20/22) of dogs fed with the high-fat diet, indicating the successful establishment of a hypertension model. The amplitude of the blood pressure increase was slightly higher than those reported in previous reports [18, 19]. Heart rates were also slightly elevated in dogs fed with the high-fat diet (*P* > 0.05).

Multiple studies on hypertension have demonstrated that increased sympathetic nerve activity plays a critical role in the pathological mechanisms of refractory hypertension. The RDN procedure developed in recent years ablates the afferent and efferent sympathetic nervous fibers located in the adventitia of the renal arteries through radiofrequency catheters, which blocks the pathways activated by renal sympathetic nerves, reduces the activity of sympathetic nerves, and eventually controls blood pressure. However, there is considerable controversy regarding the clinical efficacy of RDN. The reported clinical efficacy of RDN varies greatly between studies [9, 20]. Specifically, in the SYMPLICITY HTN-3 trial, the 6-month follow-up results failed to meet the primary endpoint of effectively reducing blood pressure levels [20]. However, some researchers have supported the notion that experimental results may be influenced by a multiplicity of practice-specific factors, such as a short learning curve and follow-up time, as well as defects in the design of surgical instruments [9, 20]. Therefore, as a new technology, RDN requires further exploration and improvement. In this study, the blood pressure levels of the experimental animals were strictly monitored. The SBP and DBP levels were statistically decreased in the RDN group at 1, 3, and 6 months after the RDN procedure as compared with the SBP and DBP levels before RDN and in the sham-operated group at the same time points (*P* < 0.05). At 6 months after RDN, dogs in the RDN group maintained near-normal blood pressure levels. By contrast, the blood pressure levels were statistically increased in the sham-operated group relative to the control group. The results indicated that RDN had a statistical antihypertensive effect that was stable and long lasting. The results were similar to that of Dijk et al., and in spontaneous hypertension rats, the progressions of renal injury, cardiac remodeling, and increased blood pressure were regulated by renal sympathetic activities because they were inhibited by the RDN procedure [21]. Selejan et al. showed that sympathetic activity suppressed sRAGE/RAGE balance through the sympathetic modulation accomplished by the RDN procedure and thus prevented RAGE-induced cardiac lesions in subjects with hypertension and metabolic syndrome [22]. However, unlike the existing literature, this study uses Beagle dogs as target animals to further verify the application of RDN in a large-animal model. Comparing the body weights before and after the RDN procedure, the RDN group and the sham-operated group exhibited insignificantly increased body weights after RDN treatment (*P* > 0.05). The results may be related to the continued high-fat feeding after RDN. No statistical difference was found in body weights between the RDN group and sham-operated group at 6 months after RDN. Therefore, the effect of body weight on blood pressure can be excluded. We also compared heart rates before and after the RDN procedure and found that heart rate was reduced in the RDN group at various time points after RDN. In addition, the heart rate was slightly lower in the RDN group relative to the sham-operated group. Standard radiofrequency catheters were used in the present study. No substantial differences exist between the catheters used in the present study and Simplicity catheters (Medtronic, Inc.) in terms of catheter material, radiofrequency energy source, and mode of ablation. The main differences lie in the length of the ablation electrode at the head portion of the catheter and the shape of the distal end of the catheter. The length of the ablation electrode on the standard radiofrequency catheter was 4 mm, which ensures an increase in the contact area with the renal artery, as well as the ablation and injury of additional renal sympathetic nerves. The employment of the standard radiofrequency catheter is conducive to controlling the ablation temperature, reducing renal artery intimal injury, and increasing the safety of the procedure. The safety and effectiveness of the standard radiofrequency catheters have been confirmed by our previous investigation and studies conducted by other researchers [15, 23, 24].

During the RDN procedure, the ablation power was set to 10 W, which was higher than the power recommended for Simplicity catheters. The pathological results of our previous animal study showed that the application of an ablation power of 8 W induced minimal renal nerve damage and a poor antihypertensive effect (data not shown). The reason for the phenomenon may be that the length of the ablation electrode on the standard radiofrequency catheter was 4 mm. The standard radiofrequency catheter offered a larger effective ablation area and dissipated heat more quickly. Therefore, a power increase is necessary to effectively damage the renal sympathetic nerves in the adventitia of the renal arteries. A better ablation catheter should be designed to increase the effect of RDN. In the present study, ten beagles developed subcutaneous hematomas after the RDN procedure, which was related to the extensive femoral artery injury caused by the 8F arterial sheath, which had a large diameter, and inadequate hemostatic press after RDN. However, after reapplying the hemostatic bandage, the dogs all recovered within 1 week. At 1, 3, and 6 months after the RDN procedure, the experimental dogs were reexamined and subjected to renal arteriography. Long-term complications, such as renal artery stenosis, renal artery dissection, and aneurysm, were not detected. These findings were consistent with the results of RDN-related clinical trials and supported the safety of RDN [9, 20].

The major left ventricular structural and functional changes caused by hypertension are LVH and myocardial fibrosis. Evidence shows that an increase in the LVM precedes the onset of overt hypertension [25, 26]. Left ventricular hypertrophy can be diagnosed via an electrocardiogram (ECG) or color Doppler echocardiography [25, 26]. Electrocardiogram diagnoses mild LVH at a rate that is 7-35% lower than echocardiography and diagnoses moderate-to-severe LVH at rates 10-50% lower than echocardiography [25]. At present, LVH is defined as an increase in LVM, which can be expressed as ventricular wall thickening, cardiac chamber enlargement, or both. Hypertension induces an increase in LVM mainly through a chronic increase in left ventricular afterload, neuroendocrine activation, and genetic factors. The pathophysiological mechanisms of LVH involve a statistical increase in the number and size of sarcomeres in every cardiomyocyte. In addition, hypertension may lead to myocardial interstitial fibrosis [27]. Both LVH and myocardial fibrosis increase left ventricular stiffness, resulting in left ventricular diastolic dysfunction. The present study showed that LVM and LVMI were statistically increased, while peak E/A ratio was markedly reduced, in the RDN group and sham-operated group after the establishment of the model relative to baseline and the control group, indicating that LVH and diastolic dysfunction occurred early in dogs with hypertension. However, at 6 months after the RDN procedure, the LVM and LVMI were statistically decreased in the RDN group relative to the sham-operated group, whereas the peak E/A ratio was drastically increased, which was consistent with the results of Jiang et al. [28]. The results indicated that RDN alleviated LVH and improved diastolic function. In their study, the RDN procedure was performed in spontaneous hypertension rats, and the results showed that blood pressure and LVMI were statistically reduced after the surgery. The results indicated that RDN alleviated LVH and improved diastolic function. In addition, H&E staining of the left ventricular myocardium showed that the size and cross-sectional area of the cardiomyocytes were statistically smaller in the RDN group relative to the sham-operated group. The results further confirmed that the RDN procedure alleviated LVH from the histopathological perspective.

Myocardial fibrosis refers to the excess accumulation of collagen fibers, the statistical elevation of collagen concentration, or a change in the collagenous components of the normal tissue structure of the myocardium. In clinical practice, collagen-specific staining is often performed on endomyocardial biopsy or autopsy tissues, and the CVF and PVCA are often calculated to evaluate the proportion and extent of interstitial fibrosis and perivascular fibrosis [29]. In the present study, the Masson staining of the myocardium showed that myocardial fibrosis occurred in both the RDN group and the sham-operated group. However, CVF and PVCA were statistically lower in the RDN group relative to the sham-operated group (*P* < 0.05). The results further demonstrated that the RDN procedure alleviated left ventricular myocardial fibrosis from a histopathological perspective.

A great deal of studies have shown that AngII plays a vital role in the development of LVH in patients with hypertension [30, 31]. Research has revealed that, in transgenic rats exhibiting the overexpression of the angiotensinogen gene, the contributing role of AngII in the development of myocardial hypertrophy appears to be independent of hypertension [32]. However, it is unclear whether the renin-angiotensin-aldosterone system (RAAS) regulates the production of Ang II through *in situ* renin release or the uptake of circulating renin [33]. Regression analysis has shown that plasma AngII is statistically correlated with LVM and that this correlation is independent of blood pressure and body size [34, 35]. The direct role of increased levels of NE on the human heart beyond systemic BP changes in humans has been shown most convincingly with a radiotracer dilution methodology in hypertensive subjects [36]. The RAAS is also closely related to myocardial fibrosis. AngII promotes the synthesis of stromal collagen by cardiac fibroblasts. The mechanism by which AngII induces myocardial fibrosis involves the binding of AngII to Ang II type 1 (AT1) receptor. The binding of AngII to the AT1 receptor activates extracellular signal-regulated kinase (ERK) through tyrosine kinase pathways; induces fibroblast proliferation; and activates the expression of collagen, fibrin, and integrin genes, thereby inducing myocardial fibrosis [37].

The present study showed that, after the successful establishment of the hypertension model, the serum NE and AngII levels were statistically higher in the RDN group and sham-operated group relative to the control group. The results indicated that the increased activities of the sympathetic nerves and RAAS represented an important mechanism underlying high-fat diet-induced hypertension. After the RDN procedure, the serum level of NE and AngII was markedly reduced in the RDN group relative to the sham-operated group, indicating that RDN successfully reduced the activity of sympathetic nerves and the RAAS. Specifically, we examined, for the first time, local NE and AngII levels in myocardial tissues at 6 months after RDN and found that the local NE and AngII levels in the left ventricular myocardium were significantly reduced in the RDN group relative to the sham-operated group. The results indicated that RDN reduced the level of NE and AngII in the circulation and myocardial tissues by decreasing the activity of the sympathetic nerves and RAAS, thereby reducing the direct injury to cardiomyocytes, mediated by NE and AngII. In addition to lowering blood pressure, reducing NE and AngII levels may represent another important mechanism by which RDN protects left ventricular structure and function. Research shows that Ang II can increase the activities of the sympathetic nervous system at various levels by facilitating NE release [38, 39]. Chen et al. also showed that RDN alleviates the onset of heart failure by downregulating angiotensin levels [40]. A study of an animal model of abdominal aortic coarctation revealed that blood pressure is not affected after sympathectomy, whereas LVH is statistically deduced, indicating that the anti-LVH effect of sympathectomy is independent of blood pressure [41]. Current clinical research data also suggest that RDN reduces LVH and improves diastolic function, independent of the blood pressure-lowering effect [13]. Therefore, we have reason to believe that RDN alleviates LVH and fibrosis by reducing NE and AngII levels in the circulation and myocardial tissues and that the above effects on the part of RDN are also independent of its blood pressure-lowering effect. The present study provides important large-animal experiment-derived preclinical evidence for RDN application to treat hypertension complicated by left ventricular remodeling.

One limitation of this study was the small sample size. Due to the sample size, we were not able to sacrifice the animals at 1 month and 3 months after the RDN procedure to examine structural changes in cardiac tissues. Therefore, it is impossible to compare the cardiac structure at the above time points with that at 6 months after RDN. In addition, the present study failed to further investigate the relationship between NE/AngII levels and hypertension-induced cardiac damage from the prospective of molecular mechanisms. In addition, the NE content in the denervated kidneys was not measured to assess the difference between the denervated kidney and the sham-operated group or the effect of RDN on glucose and other glucose metabolism parameters. The above issues will be addressed in our future studies. We will attempt to further clarify the potential mechanisms of action of RDN, develop and improve this novel treatment method, and provide new ideas about clinical applications of RDN.

## Figures and Tables

**Table 1:** Changes in body weight, heart rate, blood pressure, blood lipid levels, and renal function in experimental dogs (control group (*n* = 20), sham-operated group (*n* = 19), and RDN group ($n=19,\bar{x}\pm S$)).

Group	BW (kg)	HR (times/min)	SBP (mmHg)	DBP (mmHg)	TC (mmol/l)	TG (mmol/l)	LDL (mmol/l)	Cr (mg/dl)	CC (mmol/l)
Control group									
Baseline	12.3 ± 1.9	132.7 ± 23.9	125.7 ± 6.0	73.6 ± 6.0	5.12 ± 0.82	0.44 ± 0.12	0.11 ± 0.03	55.83 ± 10.08	0.21 ± 0.03
Preprocedure	13.1 ± 1.2	124.3 ± 6.0	129.7 ± 6.8	75.3 ± 10.6	4.80 ± 0.57	0.47 ± 0.20	0.09 ± 0.03	52.83 ± 15.22	0.20 ± 0.03
1 month	13.2 ± 1.1	122.9 ± 17.61	130.8 ± 6.5	77.3 ± 13.4				50.33 ± 7.58	0.16 ± 0.04
3 months	13.3 ± 1.0	125.4 ± 18.26	130.8 ± 6.5	81.6 ± 4.6				48.00 ± 8.22	0.17 ± 0.03
6 months	13.3 ± 0.8	124.3 ± 21.57	127.8 ± 3.6	78.2 ± 7.4	4.95 ± 0.47	0.49 ± 0.14	0.10 ± 0.04	54.00 ± 2.28	0.18 ± 0.03
Sham-operated group									
Baseline	12.3 ± 0.5	129.6 ± 11.7	124.7 ± 8.2	76.4 ± 8.4	5.41 ± 0.81	0.46 ± 0.09	0.10 ± 0.02	57.75 ± 6.75	0.21 ± 0.03
Preprocedure	17.5 ± 2.7^ab^	141.3 ± 13.5	151.6 ± 12.3^ab^	95.1 ± 9.2^ab^	7.73 ± 1.20^ab^	0.56 ± 0.18^ab^	0.21 ± 0.08^ab^	52.37 ± 9.05	0.20 ± 0.05
1 month	17.6 ± 2.9^ab^	136.5 ± 8.72	153.7 ± 13.7^ab^	93.2 ± 12.1^ab^				51.50 ± 7.91	0.18 ± 0.05
3 months	18.3 ± 1.8^ab^	140.5 ± 17.17	152.5 ± 12.6^ab^	91.6 ± 9.7^ab^				53.25 ± 5.57	0.19 ± 0.04
6 months	18.5 ± 1.9^ab^	143.9 ± 18.01	155.8 ± 8.5^ab^	95.8 ± 16.0^ab^	7.95 ± 1.14^ab^	0.61 ± 0.15^ab^	0.20 ± 0.05^ab^	54.25 ± 3.69	0.20 ± 0.03
RDN group									
Baseline	11.8 ± 0.8	131.5 ± 10.7	123.7 ± 10	77.1 ± 9.6	5.41 ± 0.81	0.50 ± 0.13	0.12 ± 0.05	53.33 ± 12.32	0.21 ± 0.05
Preprocedure	17.6 ± 2.0^ab^	143.7 ± 14.2	153.1 ± 9.5^ab^	92.5 ± 7.6^ab^	7.84 ± 0.88^ab^	0.56 ± 0.12^ab^	0.23 ± 0.06^ab^	47.22 ± 12.39	0.21 ± 0.05
1 month	17.7 ± 2.1^ab^	133.3 ± 12.76	134.6 ± 8.4^cd^	82.9 ± 7.0^cd^				46.22 ± 6.94	0.16 ± 0.04
3 months	17.9 ± 2.4^ab^	131.4 ± 15.08	131.4 ± 10.4^cd^	82.9 ± 7.0^cd^				47.00 ± 7.48	0.17 ± 0.03
6 months	18.6 ± 2.3^ab^	131.2 ± 22.18	129.6 ± 8.4^cd^	79.4 ± 5.3^cd^	7.23 ± 1.27^ab^	0.56 ± 0.13^ab^	0.18 ± 0.07^ab^	50.44 ± 4.58	0.18 ± 0.03

^a^Compared with baseline, *P* < 0.05. ^b^Compared with the control group, *P* < 0.05. ^c^Compared with the sham-operated group, *P* < 0.05. ^d^Compared with preprocedure, *P* < 0.05. BW: body weight; HR: heart rate; SBP: systolic blood pressure; DBP: diastolic blood pressure; TC: total cholesterol; TG: triglycerides; LDL: low-density lipoprotein; Cr: creatinine; CC: cystatin C.

**Table 2:** Changes in levels of norepinephrine (NE) and angiotensin II (AngII) in experimental dogs (control group (*n* = 20), sham-operated group (*n* = 19), and RDN group ($n=19,\bar{x}\pm S$)).

Group	NE (pg/ml)	AngII (pg/ml)	Myocardial NE (pg/mg)	Myocardial AngII (pg/mg)
Control group				
Baseline	123.36 ± 80.16	66.48 ± 5.19		
Preprocedure	141.13 ± 52.17	67.84 ± 8.03		
6 months	180.82 ± 77.03	69.33 ± 2.58	1953.5 ± 606.7	963.0 ± 192.2
Sham-operated group				
Baseline	140.16 ± 70.63	63.40 ± 8.80		
Preprocedure	553.2 ± 202.90^ab^	130.40 ± 8.3^ab^		
6 months	609.20 ± 144.36^ab^	136.20 ± 6.27^ab^	4343.6 ± 346.4^b^	1217.1 ± 186.9^b^
RDN group				
Baseline	106.12 ± 52.01	63.31 ± 7.19		
Preprocedure	531.07 ± 159.58^ab^	122.74 ± 10.78^ab^		
6 months	299.45 ± 97.08^acd^	88.49 ± 5.69^acd^	2272.9 ± 774.0^c^	1077.8 ± 196.0^c^

^a^Compared with baseline, *P* < 0.05. ^b^Compared with the control group, *P* < 0.05. ^c^Compared with the sham-operated group, *P* < 0.05. ^d^Compared with preprocedure, *P* < 0.05.

**Table 3:** Echocardiographic evaluation of structural cardiac changes in experimental dogs (control group (*n* = 20), sham-operated group (*n* = 19), and RDN group ($n=19,\bar{x}\pm S$)).

Group	LVEDD (mm)	IVST (mm)	LVPW (mm)	LVM (g)	LVMI (g/kg)	LVEF (%) (cm/s)	Peak E (cm/s)	Peak A (cm/s)	Peak E/A	LAD (mm)
Control group										
Baseline	25.7 ± 1.0	6.33 ± 0.5	6.0 (5.0-7.0)	30.7 ± 3.4	2.49 ± 0.1	61.2 ± 2.2	0.68 ± 0.2	0.52 ± 0.1	1.32 ± 0.1	19.8 ± 1.8
Preprocedure	25.3 ± 1.4	6.17 ± 0.4	6.0 (6.0-7.0)	30.9 ± 2.8	2.48 ± 0.2	60.3 ± 5.9	0.74 ± 0.1	0.63 ± 0.1	1.17 ± 0.3	21.0 ± 1.6
6 months	25.6 ± 1.5	6.0 ± 0.6	6.0 (6.0-7.0)	32.2 ± 2.8	2.35 ± 0.1	62.8 ± 5.0	0.79 ± 0.1	0.66 ± 0.1	1.20 ± 0.1	20.6 ± 1.5
Sham-operated group										
Baseline	25.6 ± 1.0	6.50 ± 0.5	6.0 (6.0-7.0)	31.9 ± 4.2	2.57 ± 0.3	61.6 ± 1.9	0.68 ± 0.1	0.51 ± 0.1	1.31 ± 0.1	19.5 ± 1.1
Preprocedure	28.6 ± 1.3^ad^	9.88 ± 0.6^ad^	8.0 (7.0-8.0)^ad^	62.8 ± 5.9^ad^	3.62 ± 0.4^ad^	64.2 ± 3.6	0.54 ± 0.1^ad^	0.59 ± 0.1	0.91 ± 0.1^ad^	20.5 ± 1.3
6 months	28.1 ± 1.1^ad^	10.0 ± 0.6^ad^	8.0 (7.0-9.0)^ad^	65.1 ± 4.3^ad^	3.63 ± 0.6^ad^	61.8 ± 4.1	0.48 ± 0.1^ad^	0.55 ± 0.1^ad^	0.87 ± 0.4^ad^	25.3 ± 1.6^ad^
RDN group										
Baseline	25.1 ± 1.0	6.64 ± 0.5	6.0 (5.0-7.0)	32.0 ± 4.5	2.69 ± 0.2	62.0 ± 1.9	0.68 ± 0.1	0.51 ± 0.1	1.27 ± 0.1	19.8 ± 1.4
Preprocedure	29.1 ± 1.0^ad^	10.0 ± 0.7^ad^	7.0 (7.0-8.0)^ad^	62.3 ± 5.2^ad^	3.57 ± 0.5^ad^	62.0 ± 2.2	0.48 ± 0.1^ad^	0.57 ± 0.1	0.83 ± 0.1^ad^	20.5 ± 1.2
6 months	29.7 ± 1.9^ad^	8.6 ± 0.8^bc^	6.1 (6.0-7.0)^bc^	52.2 ± 4.4^bc^	2.82 ± 0.1^bc^	62.3 ± 3.1	0.70 ± 0.1^bc^	0.56 ± 0.1	1.25 ± 0.1^bc^	21.1 ± 1.2

^a^Compared with baseline, *P* < 0.05. ^b^Compared with preprocedure, *P* < 0.05. ^c^Compared with the sham-operated group, *P* < 0.05. ^d^Compared with the control group, *P* < 0.05. LVEDD: left ventricular end-diastolic diameter; IVST: interventricular septal thickness; LVPW: left ventricular posterior wall; LVM: left ventricular mass; LVMI: left ventricular mass index; LVEF: left ventricular ejection fraction; peak E: early peak diastolic velocity; peak A: peak atrial velocity; peak E/A: the ratio of peak E to peak A; LAD: left atrial diameter.

**Table 4:** Changes in collagen volume fraction (CVF) and perivascular circumferential collagen area (PVCA) in the left ventricular myocardium in experimental dogs ($\bar{x}\pm S$).

Group	Parameters	
	CVF (%)	PVCA
Control (*n* = 10)	2.241 ± 0.67	0.67 ± 0.11
Surgically treated (*n* = 10)	4.32 ± 1.33^a^	0.85 ± 0.16
Sham-operated (*n* = 10)	12.65 ± 1.67^ab^	1.11 ± 0.19^ab^

^a^Compared with the control group, *P* < 0.05. ^b^Compared with the RDN group, *P* < 0.05.

## Data Availability

All data generated or analyzed during this study are included in this article. The datasets used and/or analyzed during the current study are available from the corresponding author on reasonable request.

## Abbreviations

AngII: Angiotensin II
CVF: Collagen volume fraction
DBP: Diastolic blood pressure
EF: Ejection fraction
HE staining: Hematoxylin-eosin staining
IVSTd: Interventricular septal depth
LAD: Left atrial diameter
LVEDD: Left ventricular end-diastolic diameter
LVEF: Left ventricular ejection fraction
LVH: Left ventricular hypertrophy
LVM: Left ventricular mass
LVMI: Left ventricular mass index
LVSDD: Left ventricular end-systolic diameter
LVPWT: Left ventricular posterior wall depth
NE: Norepinephrine
SBP: Systolic blood pressure
RAAS: Renin-angiotensin-aldosterone system
RDN: Renal sympathetic denervation
PVCA: Perivascular collagen area.
